# Cutaneous Leishmaniasis in a Non-endemic Area in Mexico

**DOI:** 10.7759/cureus.38228

**Published:** 2023-04-27

**Authors:** Mayte Aseret Martinez Niño, Juan R Camacho Galván, Urania del Rocio Castillo Cruz, Gabriela Perez-Coronado

**Affiliations:** 1 General Medicine, Instituto Tecnológico y de Estudios Superiores de Monterrey, Monterrey, MEX; 2 Internal Medicine, Universidad Autonoma de Coahuila, Torreón, MEX; 3 Internal Medicine, Instituto Mexicano del Seguro Social, Torreón, MEX; 4 Dermatology, Instituto Mexicano del Seguro Social, Saltillo, MEX

**Keywords:** new world leishmaniasis, leishmania mexicana, visceral leishmaniasis (vl), muco-cutaneous leishmaniasis, cutaneous leishmaniasis

## Abstract

Leishmaniasis is a zoonotic disease caused by a parasite of the genus Leishmania endemic in 102 countries around the world. Clinical features can be classified as cutaneous, mucocutaneous, and visceral leishmaniasis; this will depend on the species of leishmania responsible for the disease and the immunologic response of the host. We present the case of a 14-year-old male who started with a small papular lesion in the helix of the left ear, which later spread to the rest of the helix, tragus, and part of the auricular concha. On physical examination, an erythematous plaque with a scale that deforms the auricular surface was observed. In histopathology, acute and chronic inflammatory processes were observed with an accumulation of macrophages that contain amastigotes inside (Leishman Donovan bodies). The diagnosis of leishmaniasis represents a challenge in non-endemic regions. It is important to ask about travel history to endemic areas and perform assays to confirm the diagnosis and initiate treatment to prevent mortality and morbidity.

## Introduction

Leishmaniasis is a vector-borne disease caused by a parasite of the genus Leishmania, transmitted to humans through the bite of the female sandfly Lutzomyia and Phlebotomus [1,2]. Leishmaniasis is endemic in 102 countries, with about 1.3 million new cases, and 20,000 to 30,000 deaths reported every year. It is estimated that worldwide there are 12 to 15 million people infected and 350 million people at high risk of acquiring the disease [2,3]. The three clinical syndromes that can develop with the infection are cutaneous, mucocutaneous, and visceral leishmaniasis. Worldwide, the most common clinical presentation is cutaneous leishmaniasis [4]. The severity and clinical manifestations will depend on the species of leishmania responsible for the disease and the host's immunologic response ranging from self-healing and painless cutaneous ulcer to severe life-threatening disease. In Mexico, the endemic areas are the southeast states of the country like Veracruz, Tabasco, Oaxaca, Chiapas, Yucatán, Quintana Roo, and Campeche [5]. This article was presented as a poster in the “Congreso Conmemorativo de Dermatología Pediátrica” in Guadalajara, México. 

## Case presentation

**Figure 1:**
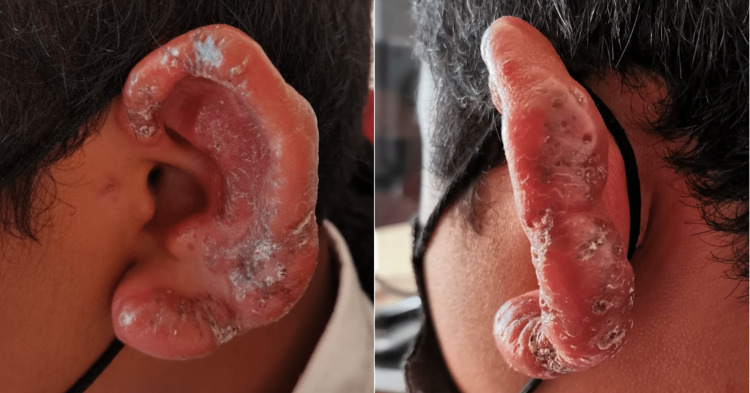
Scale-covered erythematous plaque with deformity of the atrial surface

**Figure 2:**
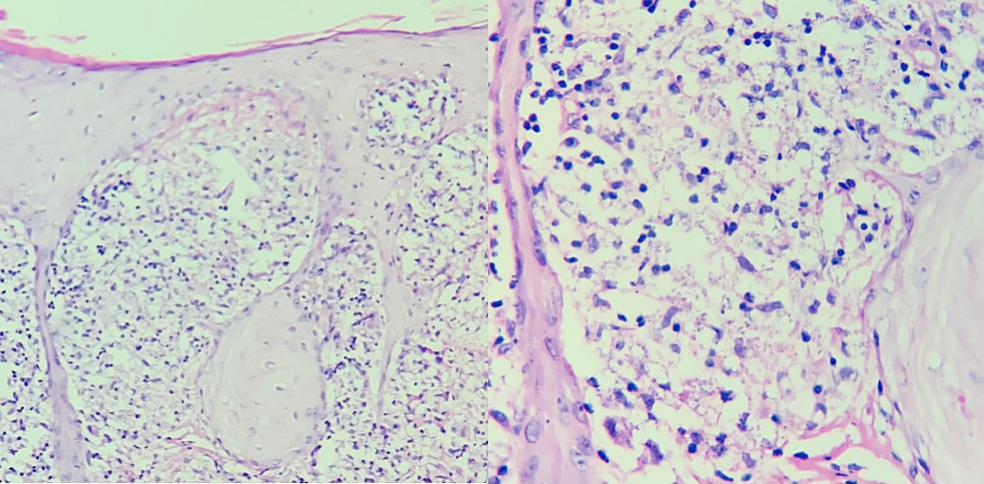
Pseudoepitheliomatous hyperplasia and dermis with macrophages containing amastigotes inside

**Figure 3:**
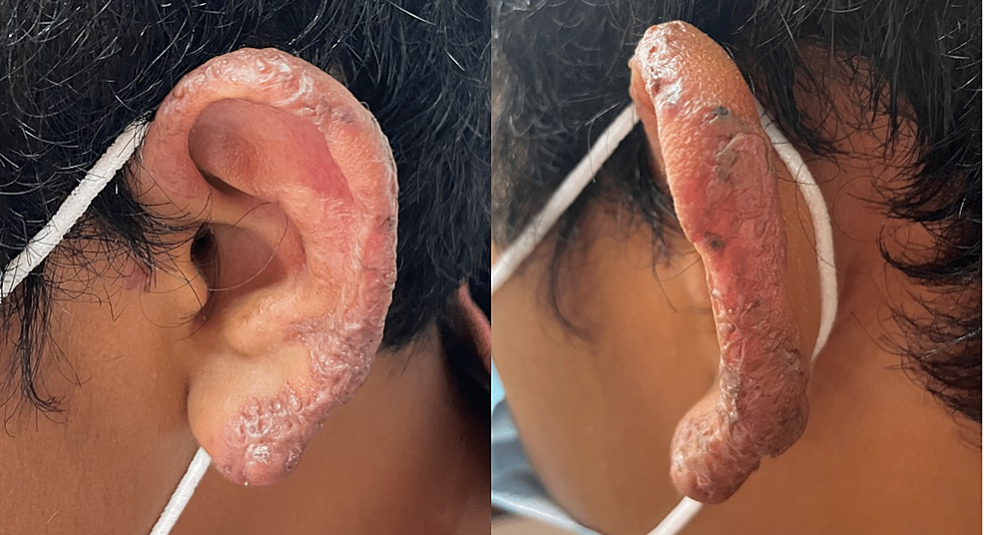
Scale and residual hyperpigmentation at the end of treatment

A 14-year-old male, originally from Veracruz, México, who has lived in Coahuila, México for 10 months observed a small papular lesion in the helix of the left ear one year ago, which later spread to the rest of the helix, tragus, and part of the auricular concha. He presented to the dermatology department with an erythematous plaque with scale that deforms the auricular surface (Figure 1). On histopathology, an acute and chronic inflammatory process, pseudoepitheliomatous hyperplasia, and accumulation of macrophages that contain amastigotes inside (Leishman Donovan bodies) were observed (Figure 2). The treatment was started with intramuscular meglumine antimoniate 1.5 g for 25 days. At the end of the treatment, the patient showed notable improvement, presenting only slight scaling and residual hyperpigmentation (Figure 3).

 

## Discussion

Worldwide, the number of cases of leishmaniasis has been increasing due to diverse factors such as climate change, geographic expansion of parasites, vectors, and reservoirs, immunosuppression by organ transplant, immunotherapy or HIV, migration, and increased travel to endemic areas [1-3,6,7].

We describe a case of cutaneous Leishmaniasis in a northern state of Mexico. Although the southern states of Mexico are the endemic areas for Leishmaniasis, sporadic cases have been reported in non-endemic areas of the country according to reports of the INDRE [5]. Some authors have reported isolated cases in non-endemic areas due to activities related to migration and tourism. Domínguez-Ugalde et al. reported a case of cutaneous leishmaniasis in a patient in Mexico City, who had previously lived in Oaxaca, Mexico, one of the states with the highest prevalence of Leishmaniasis [8]. Siller et al. described the case of cutaneous leishmaniasis in San Diego, California, in a male patient after a trip to Yucatan, Mexico [9]. 

More than 20 different Leishmania species can cause the disease. Worldwide distribution can be classified as Old World leishmaniasis caused by the species L. tropica, L.major, L. aethiopica, L. donovani, and L. infantum and New World leishmaniasis caused by L. mexicana, L. braziliensis, L. guyanensis, L. amazonensis, L. chagasi, and L. naiffi [4,10].

Cutaneous leishmaniasis can be divided into localized or diffuse and is frequently caused by L. tropica, L. major, L aethiopica, L. mexicana, and L. braziliensis. Localized cutaneous leishmaniasis mainly occurs in exposed areas of the body accessible to the bite of the sandflies like the face (especially in the ears, nose, and cheeks), neck, and limbs. The incubation period usually ranges from one to four weeks, but it can take up to three years. The typical presentation also known as the “chiclero’s ulcer” is characterized by an asymptomatic papule that enlarges and turns into a well-defined painless ulcer with thick borders [1,10]. In most cases of cutaneous leishmaniasis, the lesions will show spontaneous healing in 2 to 18 months; however, some lesions can last up to 20 years leading to scarring and deformation [1,6]. Diffuse cutaneous leishmaniasis manifests as asymptomatic nodules that progress until they spread to other areas of the cutaneous surface and mainly affect the face, ears, elbows, and knees. It is estimated that 30% of the cases will develop invasion of the oral mucosa and nasopharynx.

Mucocutaneous leishmaniasis can present simultaneously with skin lesions or appear after the resolution of the cutaneous lesions. The route of dissemination can be lymphatic or hematogenous. The most frequently involved agent is L. Braziliensis, but also L. panamensis, L. amazonensis, and L. guyanensis can be involved [10]. It is characterized by lesions that destroy the nasal and oral cavities and can extend to the oropharynx and trachea. Risk factors for developing mucocutaneous leishmaniasis are immunocompromised patients, multiple or large skin lesions, and persistence or absence of healing of the primary skin lesion. This clinical presentation of the disease can be life-threatening and can leave permanent facial disfigurement [6].

Visceral leishmaniasis or kala-azar is the most serious form of the disease and can be life-threatening. Clinical presentation includes fever, splenomegaly, hepatomegaly, lymphadenopathy, pancytopenia, hypergammaglobulinemia, and weight loss [1,6]. If treatment is not started, the disease will progress causing hemorrhage, secondary infections, and multisystem failure leading to death [11]. The main species involved are L. donovani, L. infantum, and L. chagasi [10]. The countries with the highest incidence of visceral leishmaniasis are Ethiopia, India, Brazil, Somalia, Kenya, Sudan, and South Sudan. It is estimated that more than 90% of all cases of visceral leishmaniasis occur in these countries. [6]

Clinical manifestations and travel history to endemic areas must be considered for diagnosis [1,12]. There is no gold-standard test for the diagnosis and clinical practice guidelines recommended to use multiple diagnostic methods [4,13].

The diagnostic methods that can be used include PCR which is considered the most sensitive test, examination of a smear tissue under a microscope which is easy, cheap, and quick to perform, histologic examination with Giemsa or hematoxylin-eosin stain, culture in Novy-McNeal-Nicolle medium (N-N-N medium), Montenegro test, and determination of anti-K39 antibodies and anti-Leishmania IgG in serum [1,6,13]. For visceral leishmaniasis, a biopsy must be obtained from the spleen, bone marrow, or lymph node to observe the protozoans through a microscope [1].

Histological findings in the early stage of the disease include hyperplasia and ulceration of the epidermis, dense and diffuse infiltrate mainly of macrophages, but lymphocytes and plasma cells can also be seen. The amastigotes (Leishman Donovan bodies) are round or oval microorganisms of approximately 1 to 4 μm length that can be identified within macrophages through a microscope [6,12,13]. In the late stages of the disease, the number of macrophages and amastigotes decreases, and plasma cells and tuberculoid granuloma predominate [12,14].

Several therapeutic options have been proposed and for many years pentavalent antimonials, such as sodium stibogluconate and meglumine antimoniate have been considered first-line drugs; however, the only FDA-approved drugs are intravenous liposomal amphotericin B and oral miltefosine [4,12,15]. 

Treatment always should be individualized for each patient. For mucocutaneous and visceral leishmaniasis, systemic therapy is recommended, for cutaneous leishmaniasis systemic treatment is recommended in patients with complex lesions, and local therapy is preferred in patients with simple cutaneous lesions. The guideline for the Diagnosis and Treatment of Leishmaniasis published by the Infectious Diseases Society of America (IDSA) and the American Society of Tropical Medicine and Hygiene (ASTMH) suggested a classification for cutaneous leishmaniasis that can modify and guide the treatment, classifying the cutaneous lesions as simple or complex (Table 1) [4].

**Table 1:** Clinical characteristics of simple and complex cutaneous leishmaniasis Reproduced from “Diagnosis and Treatment of Leishmaniasis: Clinical Practice Guidelines by the Infectious Diseases Society of America (IDSA) and the American Society of Tropical Medicine and Hygiene (ASTMH)” [4]

Simple Cutaneous Lesion
Caused by a Leishmania species unlikely to be associated with mucosal leishmaniasis
No mucosal involvement noted
Absence of characteristics of complex Cutaneous leishmaniasis
Only a single or a few skin lesions
Small lesion size (diameter <1 cm)
Location of lesion feasible for local treatment
Nonexposed skin (i.e, not cosmetically important)
Immunocompetent host
Lesion(s) resolving without prior therapy
Complex Cutaneous Lesion
Caused by a Leishmania species that can be associated with increased risk for mucocutaneous leishmaniasis
Local subcutaneous nodules
Large regional adenopathy
>4 skin lesions of substantial size (eg, >1 cm)
Large individual skin lesion (diameter ≥5 cm)
Size or location of lesion such that local treatment is not feasible
Lesion on face, including ears, eyelids, or lips; fingers, toes, or other joints; or genitalia
Immunocompromised host (especially with respect to cell-mediated immunity)
Clinical failure of local therapy
Unusual syndromes: leishmaniasis recidivans, diffuse cutaneous leishmaniasis or disseminated cutaneous leishmaniasis

For cutaneous leishmaniasis, local therapy available options include intralesional injections, heat and cryotherapy, and topical creams with paromomycin. Intralesional administration of 0.2-5 ml of antimonials every three to seven days can be done directly into the lesion with a total of two to five doses [4,15]. Topical combination with 15% paromomycin and 12% methylbenzethonium chloride ointment two times per day for 10 days with rest for 10 days and reapply two times per day for 10 more days, 15% paromomycin, and 0.5% gentamicin cream once per day for 20 days is administered. Heat therapy using radiofrequency directly to the skin at 50°C for 30 seconds, one session is usually enough but sometimes up to three sessions may be required. Cryotherapy with liquid nitrogen every three weeks for up to three sessions if necessary [4].

Systemic treatment for cutaneous infection includes sodium stibogluconate or meglumine antimoniate at a dose of 20 mg/kg/day intravenous or intramuscular for 20 days, amphotericin B deoxycholate intravenous at a dose of 0.5-1.0 mg/kg daily or every other day (cumulative total 15-30 mg/kg), and liposomal amphotericin B intravenous 3 mg/kg/day on days 1-5 and 10 or on days 1-7 (cumulative total 18-21 mg/kg). Oral options include miltefosine 2.5 mg/kg/day maximum of 150 mg/day for 28 days, fluconazole 200 mg/day for six weeks, and ketoconazole 600 mg/day for 28 days [4,13].

Mucocutaneous disease available options are sodium stibogluconate or meglumine antimoniate at a dose of 20 mg/kg/day for 28 to 30 days, amphotericin B deoxycholate 0.5-1.0 mg/kg daily or every other day (cumulative total of 20-45 mg/kg), liposomal amphotericin B 3 mg/kg/day (cumulative total of 20-60 mg/kg), and for oral options miltefosine 2.5 mg/kg/day maximum 150 mg/day for 28 days [4].

Options for visceral leishmaniasis are sodium stibogluconate or meglumine antimoniate at a dose of 20 mg/kg/day for 28 to 30 days, amphotericin B deoxycholate 1 mg/kg daily or every other day with a total of 15-20 doses, liposomal amphotericin B 3 mg/kg/day on days 1-5, 14, and 21 (total dose 21 mg/kg) if the patient is immunocompetent and 4 mg/ kg/day on days 1-5, 10, 17, 24, 31, and 38 (total dose 40 mg/kg) if the patient is immunosuppressed, and oral miltefosine 2.5 mg/kg/day maximum 150 mg/day for 28 days [4].

The clinical impact of the increasing incidence of leishmaniasis is alarming because it continues to be a neglected parasitic disease and represents a public health problem in many countries due to its morbidity, difficult access to health services, governments with a limited budget for health care, and limited treatment options due to cost, toxicity, and availability [5,9]. The diagnosis represents a challenge because leishmaniasis can mimic many infectious diseases and malignancies like lupus vulgaris, lepromatous leprosy, syphilis, lymphoma, Kaposi sarcoma, basal cell carcinoma, and squamous cell carcinoma, leading to diagnostic errors and unfortunate outcomes [4,12]. Patients with leishmaniasis and immunosuppression may have a more aggressive disease refractory to medical treatment [12]. It is important to highlight that patients with visceral leishmaniasis and HIV have been increasing. This represents a challenge because this co-infection results in a progression of both diseases, longer treatment with higher doses, and increased risk of death [6]. 

There are no vaccines or chemoprophylaxis available to prevent the disease; however, recommendations can be made to travelers to endemic areas such as the use of adequate clothing like pants and long-sleeved clothes, use of insect repellents, and avoiding activities with a high risk of suffering from sand fly bites [1,4].

## Conclusions

The diagnosis of leishmaniasis in non-endemic areas is complex and represents a challenge for physicians mainly because this diagnosis is only considered after previously lived or traveled to an endemic area. Worldwide, the number of cases of leishmaniasis is increasing, and this represents a serious health problem starting with healthcare providers who are not aware of this disease and difficult access to healthcare in many countries leading to misdiagnosis and inappropriate treatment. It is important to have an accurate diagnosis and appropriate treatment in order to prevent complications such as scarring, permanent disfigurement, and death. In our patient, lupus vulgaris was initially suspected because Leishmaniasis is non-endemic in the northern states of Mexico; however, with the travel history and the histopathological result, the final diagnosis was made. The patient meets the criteria of a complex lesion; a large individual skin lesion (diameter ≥5 cm) in the ear. For this reason, systemic treatment was initiated with intramuscular meglumine antimoniate, and our patient had complete resolution of the lesion thus preventing complications.
